# Isolated recurrent metastatic pulmonary nodule from the kidney: An extraordinarily long interval

**DOI:** 10.1002/rcr2.70005

**Published:** 2024-08-26

**Authors:** Cheong Kim, Hwan Jin Lee, Kyu Yun Jang, Jun Hyung Park, Jae Seok Jeong, Yong Chul Lee

**Affiliations:** ^1^ Jeonbuk National University Medical School Jeonju Republic of Korea; ^2^ Department of Internal Medicine, Research Center for Pulmonary Disorders Jeonbuk National University Medical School Jeonju Republic of Korea; ^3^ Research Institute of Clinical Medicine of Jeonbuk National University‐Biomedical Research Institute of Jeonbuk National University Hospital Jeonju Republic of Korea; ^4^ Department of Pathology Jeonbuk National University Medical School Jeonju Republic of Korea; ^5^ Respiratory Drug Development Research Institute Jeonbuk National University Medical School Jeonju Republic of Korea; ^6^ Laboratory of Respiratory Immunology and Infectious Diseases, Korea Zoonosis Research Institute Jeonbuk National University Iksan Republic of Korea

**Keywords:** long interval, nodule, recurrent

## Abstract

Our case highlights the importance of follow‐up. Previous meta‐analysis has shown that patients with sub‐centimetre nodules may have extended follow‐up intervals before requiring intervention, unlike those with larger nodules exceeding 1 cm. However, referring to our case, we can see the importance of regular and dense follow‐up.

## Graphical abstract

Diagnosis of pulmonary metastases from renal cell carcinoma (RCC) is crucial as surgical resection may result in improved survival. While previous studies have suggested that small pulmonary nodules (<1 cm) may not be associated with metastatic RCC, our case highlights the importance of vigilant monitoring and thorough evaluation of lung nodules of any size in patients with a history of RCC, even after long disease‐free intervals.

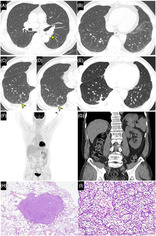

## CLINICAL IMAGE

**FIGURE 1:**
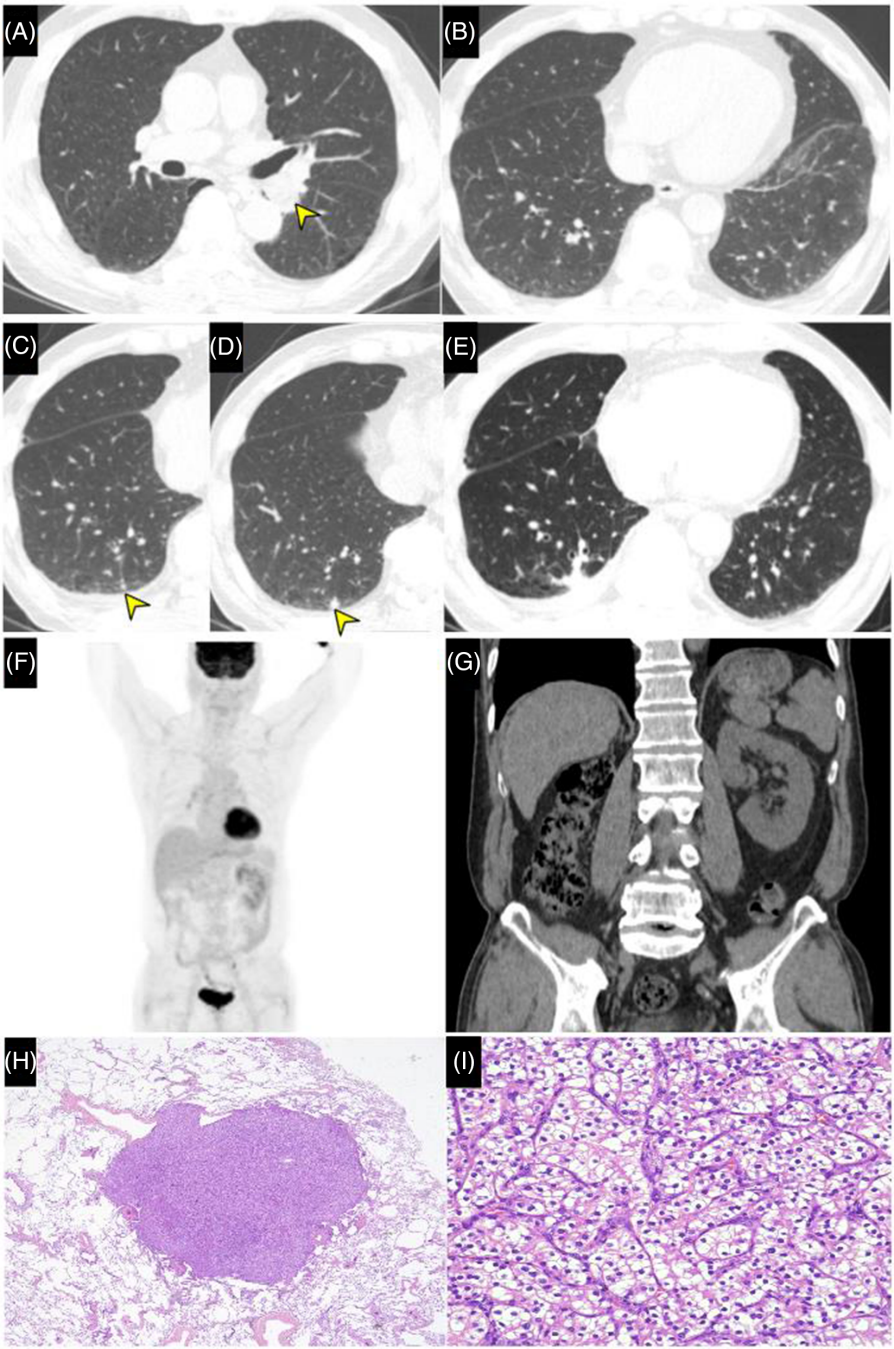
(A) High Resolution Computed Tomography (HRCT) shows a 2.5×1.7 cm oval‐shaped nodule (arrow), abutting the left lower lobar bronchus and the nodule includes internal fat attenuation. This nodule was a hamartoma and was removed through Video‐Assisted Thoracic Surgery (VATs). (B) The HRCT had been taken at the time of hamartoma removal. The HRCT indicated the absence of RCC at that time. (C) Three years after the removal of the hamartoma, a 2 mm‐sized nodule was discovered (arrow), during a follow‐up examination. There are no surgical indications, so a follow‐up was conducted. (D) One year later, a 2 mm‐sized nodule was found to have slowly grown to 6 mm (arrow). It was suspected to be a malignant tumour. (E) Video‐Assisted Thoracic Surgery (VATs) was performed, and wedge resection was carried out on the 6 mm‐sized nodule in the right lower lobe. (F) Positron Emission Tomography (PET)/CT taken before the VATs, showing a small nodule, approximately 0.7 cm in size, located in the right lower lobe, posterior basal segment, showing mild up‐take. The PET/CT also shows a right nephrectomy, and there are no abnormal hypermetabolic lesions within the abdominal cavity. (G) This is a postoperative Abdominal/Pelvic Computed Tomography (APCT) image. No evidence of cancer recurrence is observed in the left kidney on the APCT scan. Histopathological findings revealed a metastatic clear cell renal cell carcinoma (H and I). (H) Low‐power view shows a metastatic nodule in lung parenchyma (original magnification, ×20). (I) At high‐power, tumour shows characteristic histologic features of clear cell renal cell carcinoma, composed of tumour cells with clear cytoplasm arranged in a nested and tubular growth pattern (original magnification, ×400).

A 73‐year‐old male was admitted because of a slowly growing nodule discovered 1 year ago. He was a non‐smoker but had underlying mild COPD. Seventeen years ago, he underwent a right nephrectomy following a diagnosis of stage II clear cell renal cell carcinoma (ccRCC). Four years ago, he underwent wedge resection of a left lung nodule with video‐assisted thoracoscopic surgery (VATS), found to be a hamartoma (Figure 1A, arrow). At the time of resection, there were no lesions observed in the contralateral lung (Figure 1B). However, a year ago, a High‐Resolution Computed Tomography (HRCT) scan revealed a 2 mm nodule in the posterobasal segment of the right lower lobe (Figure 1C, arrow). Furthermore, in the recent HRCT, the nodule had grown to 6 mm (Figure 1D, arrow). Positron Emission Tomography (PET)/CT showed no evidence of metastasis, including the abdomen and left kidney (Figure 1F,G). We performed VATs and the nodule was confirmed as ccRCC (Figure 1H,I). A follow‐up chest CT scan 1 year later showed no signs of recurrence (Figure 1E).

Diagnosis of pulmonary metastases from RCC is crucial as surgical resection may result in improved survival.^1^ While previous studies have suggested that small pulmonary nodules (<1 cm) may not be associated with metastatic RCC,^2^ our case highlights the importance of vigilant monitoring and thorough evaluation of lung nodules of any size in patients with a history of RCC, even after long disease‐free intervals. This is particularly important considering the potential for delayed metastasis, as demonstrated some cases.^3^

## AUTHOR CONTRIBUTIONS

Cheong Kim and Hwan Jin Lee wrote the manuscript and generated figure. Kyu Yun Jang performed radiological interpretation and conducted pathological examination. Jae Seok Jeong and Yong Chul Lee directed the project. Jun Hyung Park conducted investigation. All authors critically reviewed the manuscript and approved the final version.

## FUNDING INFORMATION

This work was supported by the National Research Foundation of Korea (NRF) grant funded by the Korea government (MSIT) (No. RS‐2024‐00356349) and Special Operating Subsidy of Jeonbuk National University Industrial Cooperation Foundation. This research was supported by a grant of the Korea Health Technology R&D Project through the Korea Health Industry Development Institute (KHIDI), funded by the Ministry of Health & Welfare, Republic of Korea (grant number: RS‐2024‐00440408). This research was supported by the Bio&Medical Technology Development Program of the National Research Foundation (NRF) funded by the Korean government (MSIT) (No. RS‐2023‐00236157). This paper was supported by Fund of Biomedical Research Institute, Jeonbuk National University Hospital.

## CONFLICT OF INTEREST STATEMENT

None declared.

## ETHICS STATEMENT

The authors declare that appropriate written informed consent was obtained for the publication of this manuscript and accompanying images. The authors are accountable for all aspects of the work in ensuring that questions related to the accuracy or integrity of any part of the work are appropriately investigated and resolved. All procedures performed in this study were in accordance with the ethical standards of the institutional and/or national research committee(s) and with the Helsinki Declaration (as revised in 2013). Written informed consent was obtained from the patient for publication of this article and accompanying images. A copy of the written consent is available for review by the editorial office of this journal.

## Data Availability

None.
